# Associations between intrinsic capacity, functional difficulty, and fall outcomes among older adults in India

**DOI:** 10.1038/s41598-023-37097-x

**Published:** 2023-06-17

**Authors:** K. Muneera, T. Muhammad, Manacy Pai, Waquar Ahmed, S. Althaf

**Affiliations:** 1 National Institute of Technology, Calicut, Kerala 673601 India; 2 International Institute for Population Sciences, Mumbai, Maharashtra 400088 India; 3 Department of Sociology and Criminology, Kent State University, Kent, OH 44242 USA; 4 School of Health Systems Studies, Tata Institute of Social Sciences, Mumbai, India

**Keywords:** Geriatrics, Health policy, Epidemiology, Population screening

## Abstract

The construct of intrinsic capacity (IC) in the context of integrated care for older adults emphasizes functional assessment from a holistic perspective. It provides reliable and comparable insights on subsequent functioning and disability. Given the paucity of research on IC and health outcomes in low- and middle-income countries (LMICs), the present study examined the association of IC with geriatric conditions of functional limitations and multiple fall outcomes among older adults in India. The data used for analysis come from the first wave of the Longitudinal Aging Study in India (LASI), 2017–2018. The final sample size contains 24,136 older adults (11,871 males and 12,265 females) age 60 years or above. Multivariable binary logistic regression is employed to examine the association of IC and other explanatory factors with outcome variables of difficulty in activities of daily living (ADL) and instrumental activities of daily living (IADL), falls, fall injury, and multiple falls. Of the total sample, 24.56% of older adults were observed to be in the high IC category. The prevalence of ADL difficulty, IADL difficulty, falls, multiple falls and fall-related injury is estimated to be 19.89%, 45.00%, 12.36%, 5.49% and 5.57%, respectively. Older adults who reported high IC had a significantly lower prevalence of ADL difficulty (12.26% vs 22.38%) and IADL difficulty (31.13% vs 49.52%) than those who reported low IC. Similarly, a lower prevalence of falls (9.42% vs 13.34%), fall-related injury (4.10% vs 6.06%) and multiple falls (3.46% vs 6.16%) were reported among those who had high IC. After adjusting for a large number of confounders such as age, sex, health-related attributes and lifestyle behaviors, older adults with high IC had significantly lower odds of ADL difficulty [aOR: 0.63, CI: 0.52–0.76], IADL difficulty [aOR: 0.71, CI: 0.60–0.83], falls [aOR: 0.80, CI: 0.67–0.96], multiple falls [aOR: 0.73, CI: 0.58–0.96] and fall-related injury [aOR: 0.78, CI: 0.61–0.99]. That a high IC was independently associated with a lower risk of functional difficulty and fall outcomes in later life is of enormous value in predicting subsequent functional care needs. More specifically, the findings here imply that because regular IC monitoring can predict poor health outcomes in older adults, improvements in IC should be prioritized while formulating disability and fall prevention strategies.

## Introduction

All societies worldwide are experiencing population aging as part of the longevity revolution, with some at its earlier stages and some at more advanced stages^1^. India, now having surpassed China as the most populous country^2^, also is faced with the rapid graying of its population. This demographic transformation means rising burden of physical, mental, and cognitive diseases, and disability^3,4^, which render enormous financial health repercussions for older adults, their families, and society at large^5^.

To cope with challenges associated with population aging, the World Health Organization (WHO) developed an innovative framework of the Integrated Care for Older People (ICOPE). Two key ideas that were foundational to ICOPE include "intrinsic capacity" (IC), which is defined as the composite of an individual's physical and mental capacities that contribute to healthy aging; and "functional ability," which is the combination and interaction of IC with the social and physical environment an individual inhabits^3^. IC, which is predictive of functional potency and physiologic reserve, determines the ability to withstand stressors^6,7^. IC shifts the concept of “healthy aging” from a disease-oriented to a function-oriented approach, which creates the potential for delaying disability by introducing earlier interventions^8,9^. WHO has conceptualized IC as five interrelated domains: vitality, sensory, locomotor capacity, cognitive capacity, and psychological capacity^8,10^. In the present study, we adopt WHO terminologies of IC and functional ability, and concede that these may vary from terminologies employed in other studies.

Prior research finds that a decline in IC is strongly linked to adverse outcomes, including falls and a deterioration in activities of daily living (ADLs) and instrumental activities of daily living (IADLs)^11–15^. Additionally, a high IC score is connected with a lower likelihood of 1-year mortality^14^. Evidence also suggests that each separate component of IC is predictive of adverse health conditions among community-dwelling older adults^16–18^. For instance, sensory impairments, especially simultaneous vision and hearing deficits, have serious health implications, including compromised physical and cognitive performance^19^. Likewise, individuals with rapidly declining cognitive functioning are more likely to report worse functional health^20^. And slower gait speed has been found linked with adverse locomotive health concerns, including an increased risk of falls^21^. Additionally, balanced nutrition is needed to ensure good health and physical functioning^22^. Only a few studies, however, have analyzed the impact of five components of IC together as an independent 'emerging construct' on adverse health outcomes^11,12,14,23^.

Falls are common, constitute a major health event for older adults^24^ and are responsible for 20–30% of injuries and 50% of injury-related hospitalization^25^. Though definitions vary, a fall typically refers to an incident that results in a person unintentionally coming to rest on the ground, a floor, or other lower level^26^. A meta-analysis on the burden of falls has estimated a 31% pooled prevalence of falls among older Indians^27^. Fall-related injuries can lead to disability, dependence, institutionalization, and even premature mortality^25^. The complex convergence of cognitive, neuromuscular, sensory, and skeletal components is essential for successful ambulation. In fact, a study evaluating the predictive value of the domains of IC on the 3-year adverse health outcomes of nursing home residents revealed that a one-unit increase in the balance performance score (on a score of 0–4) and nutrition score (on a score of 0–30) reduced the risk of falling by 4%^15^.

The risk of functional difficulty is another major health concern among older adults. Loss of hearing, vision, or mobility account for a large portion of the functional difficulty in older adults^28^. ADLs and IADLs are commonly used measures of functional ability in most epidemiological and clinical research studies^29^. According to the 2011 census, in India, the prevalence of any functional difficulty was estimated to be 20.8% among the older adults^30^. Further, 24% and 48% of older adults had difficulty in ADL and IADL, respectively^31^. Research finds that measures of physical function, especially, ADL and IADL limitations often are predictive of not only physical but mental distress^32,33^. This is not surprising given that ADL and IADL related difficulty erodes autonomy, increases dependence and the likelihood of aging “out-of-place”—all of which may exacerbate mental distress^34^.

Prior studies also have examined the relevance of several socio-demographic, socioeconomic, and lifestyle factors for IC and functional health. For instance, research has observed significantly lower IC scores among individuals with comorbidity^35–37^, older age groups, women^35,38^, those who are unmarried^9^, have lower formal education^13^, and lower subjective social ranking^13^. Aside from education, household size and monthly per-capita consumption expenditure (MPCE) also are found linked to IC^9^. Moreover, gender, employment^37^, poor self-rated health, dementia^38^, and lower socioeconomic status^9^ have been documented risk factors for several measures of functional health, including falls and fall-related injuries. Lifestyle factors, namely smoking, drinking, physical exercise, and a balanced nutrition, as widely documented, remain closely connected with myriad health outcomes, including both IC^9,35,39^ and functional health^40–42^.

Several studies worldwide, most in high-income western nations and some in LMICs, have explored the concept of IC and its association with social, economic, and lifestyle factors, and a variety of health outcomes. Only one study^43^ has assessed the relevance of IC for functional decline among older adults in India. However, this study was limited to the outcomes of ADL and IADL and the sample size was small preventing the otherwise important findings of the study from being extrapolated. Our study departs from this work in that we extend the measures of functional health to include fall, multiple falls, and fall related injuries in addition to limitations surrounding ADL and IADL. We also rely on LASI, which includes a large, nationally representative sample of older Indians. A few other studies on IC, in India, that have relied on LASI or other such large scale surveys (e.g., WHO’s Sage Survey) have been limited to research on the influence of socioeconomic and lifestyle factors on IC^41,44^.

While the association between IC and functional ability has been conducted in other LMICs, findings in these studies are mixed. This generates the need for further research on the association between IC and functional ability in different countries to gauge what is uniform across nations and what is distinctive. In other words, by assessing the relevance of IC for functional resiliency in a different country with varying sociocultural, family, and financial infrastructures, research of this nature can highlight elements of the aging experience that are likely generalizable and others which may manifest out of broader macrosocial conditions specific to one country or culture, rather than aging in and of itself. A nation's favorable or unfavorable aging outcomes may thus serve as proof of the effectiveness of its social and economic policies and initiatives aimed at promoting healthy aging.

**Figure 1:**
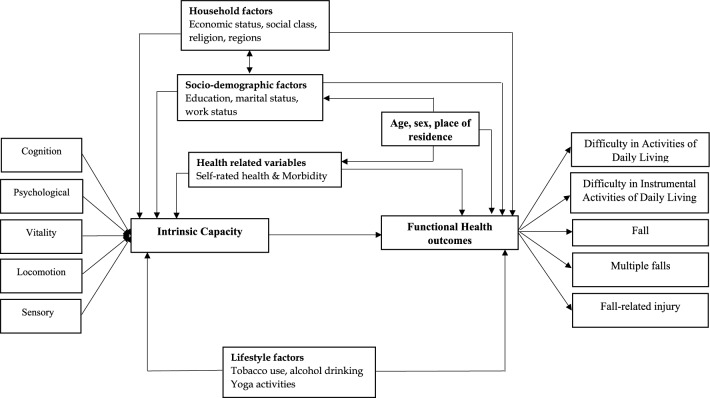
Conceptual framework of intrinsic capacity and selected adverse health outcomes.

To that end, the present study uses a large nationally representative data to examine (1) the prevalence and correlates of high IC and (2) the association of high IC and other socioeconomic and lifestyle factors with five selected geriatric conditions including ADL difficulty, IADL difficulty, falls, multiple falls, and fall-related injury among older adults in India. We hypothesize that high IC is positively associated with a significantly lower prevalence of functional difficulty and each of the fall related outcomes. Figure 1 displays the conceptual framework for the present study.

## Methods

### Data

The Longitudinal Ageing Study in India (LASI), which was conducted in 2017–2018, provided the data for the analysis. The primary goal of this survey was to examine the socioeconomic condition and health of older individuals in India. The LASI database contains crucial data on 72,250 adults in all Indian states and union territories (UT) who are 45 years of age and older, including demographics, chronic health conditions, symptom-based conditions, functional health, mental health, household economic conditions, healthcare utilisation, and health insurance. The LASI survey was carried out with the combined effort of the International Institute for Population Sciences (IIPS), Harvard T. H. Chan School of Public Health (HSPH), and the University of Southern California (USC).

A multistage stratified cluster sample design, consisting of a three-stage sampling design in rural regions and a four-stage sampling design in urban areas, is the basis for the sample selection in the LASI wave 1 study. In each state/UT, the first stage involved the selection of Primary Sampling Units (PSUs), that is, sub-districts (Tehsils/Talukas), and the second stage involved the selection of villages in rural areas and wards in urban areas in the selected PSUs. In rural areas, households were selected from selected villages in the third stage. However, sampling in urban areas involved an additional stage. Specifically, in the third stage, one Census Enumeration Block (CEB) was randomly selected in each urban area. In the fourth stage, households were selected from this CEB^45^. LASI provides survey weights at national and state levels to compensate for unequal selection probabilities at various levels of selection and to compensate for non-response. The detailed methodology, with complete information on the survey design and data collection, was published in the survey report^45^. Eligible participants of 60 years of age and above are included in the current study. After excluding the missing data for the outcome variable (n = 7328), the analysis included 24,136 older adults (11,871 men and 12,265 women) who are at least 60 years old.

### Measures

#### Outcome variable

Functional difficulty and falls were the outcome variables used in the study. The functional difficulty consists of difficulty in ADL and difficulty in IADL which have shown higher content and construct validity^46,47^. The term "ADL" describes routine daily self-care activities such as getting out of bed, walking, eating, bathing, dressing, and using the toilet (alpha = 0.87). During the interview, responses for the six items were coded as 'yes' and 'no' and older adults who struggled with any of the six activities for longer than 3 months were identified as having ADL difficulties. IADLs were tasks that are not actually related to a person's basic functioning but allow a person to live independently in a community. Respondents were asked if they were experiencing any difficulties that were anticipated to last longer than 3 months in seven activities, such as preparing a hot meal, shopping for groceries, making a phone call, taking medications, working in the garden or house, managing money, or navigating to or finding an address in strange places (alpha = 0.88). The responses to these items were coded as 'yes' and 'no' and older adults were considered to be experiencing IADL difficulty if they had trouble with any of the seven IADL activities for longer than 3 months^31^. During bivariate analysis, difficulty in ADL/IADL was also recoded into zero, one, two and three-plus according to the number of difficulties in each activities.

A fall is defined as an event that leads to someone coming to rest unintentionally on the ground, a floor, or another lower level^26^. Falls among older adults in the last 2 years were self-reported and analysed using the question,' In the past 2 years, have you fallen down?' The answers were coded as 'no' and 'yes'. While falls are typically reported using a single-year time-frame or less, the 2-year time-frame used in LASI may be associated with some recall bias on the part of the respondents. However, LASI, in which the follow-up information will be collected in each 2 years, follows other longitudinal aging studies in using the timeframe for fall outcomes such as the English Longitudinal Study of Aging (ELSA)^48^. Further, fall-related injuries were assessed by the following survey question 'In that fall, did you injure yourself seriously enough to need medical treatment?' and the responses were similarly classified as 'no' and 'yes'. Additionally, information on multiple falls was calculated from the question on the number of falls in the last 2 years. Those who reported falls more than once were considered as having multiple falls^49^.

### Explanatory variables

#### Intrinsic capacity

Based on International Classification of Functioning, Disability and Health framework, combined with available evidence, Cesari^8^ had identified the five IC domains (cognition, locomotion, sensory, vitality and psychological) as the key to managing and maintaining the IC of older adults, allowing for subsequent IC evaluation. Further, this five subfactor structure of IC was validated in multiple studies^11–13^. Similarly, the present study employed a composite IC score based on five domains consisting of nine measures. Measures were assessed using the same criteria used in a recent study in the Indian context^41^. Accordingly, a composite IC score was developed using five major domains: (i) cognition, (ii) locomotion, (iii) sensory, (iv) vitality and (v) psychological.

Cognition was assessed based on the scoring of different cognitive sub‐domains, including immediate word recall (0–10 points) and delayed word recall (0–10 points); arithmetic ability based on serial 7s (0–5 points) and backward counting from 20 (0–2 points). Out of a total score of 27, recoded as 0 if scored 0–6, considered as cognitively impaired or demented, 1 if scored 7–11, considered as mild cognitive impairment and 2 if scored 12–27, considered as normal^50^.

Locomotion was measured on the basis of walking speed/gait speed (time taken to walk a 4 m distance at the usual pace) and standing balance which is an indicator of static balance, measured progressively from semi-tandem to either side-by-side or full tandem. For assessing gait speed, respondents were asked to walk 4-m twice, and impairment was assessed by averaging the time (in seconds) taken to complete four meters (stratified by sex and height). We classified older men as having “impaired walking” if it takes seven or more seconds and six or more seconds for those with a height of 173 cm or less and a height of more than 173 cm, respectively. Similarly older women were classified with impaired walking if it takes seven or more seconds and six or more seconds by those with a height of 159 cm or less and a height of more than 159 cm, respectively. For assessing the balance, the participants were asked to hold the side of the heel of one foot, touching the toe of the other foot for a full 10 s without stepping out of place or grabbing hold of anything. If the participants were unable to hold the semi-tandem position for 10 s, they were classified into having impaired balance. Locomotion was recoded as 0, if both gait and balance were impaired, 1, if either impaired and 2 if neither impaired^51^.

The sensory domain was assessed based on impairments related to participants’ distance/near vision and hearing^14,52^. Participants were asked to rate "How good is your eyesight for seeing things at a distance/up close" on a Likert scale of 5 ranging from very good to very poor and those who rated poor or very poor were classified into having vision impairment. Similarly, information was available on whether participants were diagnosed with eye or vision problems in either or both eyes, as well as ear or hearing problems in either or both ears. Sensory was recoded as 0, if both vision and hearing impaired, 1, if either impaired and 2 if neither impaired.

The domain of vitality was measured using Body Mass Index (BMI), which refers to the weight in kilograms divided by height in meter square (kg/m^2^). BMI is considered as an indicator of the balance between energy intake and energy expenditure and a lower BMI suggests an increased risk of malnutrition according to the Malnutrition Universal Screening Tool^53^. BMI levels have been classified according to the WHO classifications: underweight ≤ 18.4; normal = 18.5 to 24.9; overweight = 25.0 to 29.9; obese ≥ 30.0. A previous study among community-dwelling older adults in China accorded a higher vitality score to older adults with a higher BMI (BMI < 20 kg/m^2^)^9^. Likewise, vitality in this study was coded as 0 if lower BMI/underweight, 1 for normal weight, and 2 for higher BMI/overweight or obese.

Psychological domain was assessed using the Self-report depression scale of the Centre for Epidemiological Scale of Depression (CES-D) with a score ranging from 0 to 30, the higher score representing higher depressive symptoms. It was recoded as 0 if scored 20–30, considered as severe symptoms, 1 if scored 10–20, considered as mild symptoms and 2 if scored 0–10, considered as no/minimal depression symptoms.

Thus, each domain was given a score of 0, 1, or 2, and the scores of five domains were added to create a composite IC score that ranged from 0 to 10, with higher values denoting greater IC. Furthermore, a recent study found that higher functional ability was associated with every standard deviation increment in the mean IC composite score^54^. Similarly, this study determined the cut-off score for high IC among older adults as one standard deviation increment (1.61) from the mean IC score (7.39) and thus, older adults who scored nine or above in the IC composite score of 0–10 were classified as having high IC.

#### Other covariates

Previous research has identified a number of determinants of functional difficulty and falls, such as socio-demographics, household factors, lifestyle factors^55^ and health-related factors^49,56^. The study used socio-demographic variables such as age (recoded as 60–69, 70–79 and 80 +), sex (male and female), education (recoded as none, primary, secondary and higher), marital status (recoded as married, widowed and others which included separated, divorced and never married) and work status (recoded as never worked, currently working, not working and retired).

The study also employed lifestyle factors such as tobacco use, episodic alcohol drinking and involvement in the yoga-related activity. The tobacco use was taken from the items (1) "Do you currently smoke any tobacco products (cigarettes, *bidis*, cigars, hookah, cheroot, etc.)?" and (2) "Do you use smokeless tobacco (such as chewing tobacco, *gutka*, *pan masala*, etc.)?" The variables were dichotomised to yes and no. Similarly, episodic alcohol drinking use was assessed with the question, "In the last 3 months, how frequently, on average, have you had at least 5 or more drinks on one occasion?" and defined as yes if the response was "1–3 days per month, 1–4 days per week, 5 or more days per week, or daily". The yoga-related activity was measured using the question, How often do you engage in the activities such as yoga, meditation, asana, pranayama or similar? The variable was dichotomised as yes (every day, more than once a week, once a week, one to three times in a month) and no (hardly ever or never). The study also used other relevant predictors. Self-rated health (SRH) was classified into good (very good, good and fair) and poor (poor and very poor). Current morbidity status was calculated based on chronic diseases such as high blood pressure, diabetes, cancer, chronic lung disease, chronic heart disease, stroke, arthritis, neurological/psychiatric issues, high cholesterol, thyroid, gastrointestinal issues, skin disease, and any other illnesses. Morbidity was coded as none, single, two and three plus.

The monthly per capita consumption expenditure (MPCE) quintile was measured using household consumption data. The details of the measure are described elsewhere^55^. The variable was then divided into five quintiles, i.e., from poorest to richest. Religion was coded as Hindu, Muslim and Others. Caste was recoded as Scheduled Caste/Scheduled Tribe (SC/ST), Other Backward Classes (OBC), and others. The other caste category refers to those having higher social status, mostly belonging to upper caste categories. The place of residence was coded as urban and rural. Also, the regions of the country were coded as North, Central, East, Northeast, West, and South.

### Statistical approach

We employed descriptive statistics and bivariate analyses to find out the preliminary results. The bivariate analysis was performed to assess the prevalence of high IC among older adults in India. Bar graphs are presented to show the distribution of older adults with high IC by sex, age, place of residence, and MPCE quintiles. Box plots are presented to show the prevalence of outcome variables by IC score across the subpopulations of male/female sexes and rural/urban residences. Chi-square tests^57^ were used to check the significance of bivariate associations.

Additionally, multivariable binary logistic regression was conducted to find out the association between the outcome variables (ADL difficulty, IADL difficulty, falls, fall injury and multiple falls), other explanatory variables and high IC. The estimates were reported in the form of adjusted odds ratios (aOR) with a 95% confidence interval (CI). All statistical models were adjusted for the selected background characteristics including age, sex, education, marital status, work status, tobacco use and alcohol consumption, yoga-related activity, self-rated health status, morbidity, MPCE quintiles, religion, caste, place of residence and regions. Based on long-standing convention^58^, P-values under 0.05 were considered statistically significant, for all the statistical tests, in this study. No multicollinearity was found among the explanatory variables used in the study models. The statistical analysis was performed using STATA version 15.1^59^. Individual survey weights were applied to account for the multi-stage stratified cluster sampling design and to provide the population level estimates. For doing so, STATA commands of *svyset* and *svy* were used in this study.

### Ethics approval and consent to participate

The study was approved by the Indian Council of Medical Research (ICMR) Ethics Committee in January 2017 and written or oral informed consent was obtained from the participants. All methods were carried out in accordance with relevant guidelines and regulations and in accordance with the World Medical Association Declaration of Helsinki.

## Results

Table 1 shows the sample distribution and prevalence of high IC among older adults by background characteristics. A total of 24.56% of older adults had a high IC in this study. A greater proportion of the sample was constituted by older adults aged 60–69 years (62.30%), women (51.49%), individuals having primary or no education (72.63%), not currently working (33.92%), middle-income category (20.70%), OBC (46.14%), Hindu (83.13) and those who were residing in rural areas (71.39%).

**Table 1:** Sample distribution by background characteristics.

Variables	Distribution	
	Frequency	w col%
High IC		
No	17,764	75.44
Yes	6372	24.56
Age (in years)		
60–69	15,408	62.30
70–79	6752	28.92
80 +	1976	8.79
Sex		
Male	11,871	48.51
Female	12,265	51.49
Marital status		
Currently in union	15,844	63.75
Widowed	7692	34.18
Others	600	2.07
Educational status		
No/primary	17,052	72.63
Secondary	4994	19.21
Higher	2090	8.16
Work status		
Never worked	6575	25.92
Not working	8017	33.92
Working	7424	32.66
Retired	2120	7.50
MPCE quintile		
Poorest	4833	20.02
Poorer	4921	20.39
Middle	4997	20.70
Richer	4858	20.13
Richest	4527	18.76
Caste		
SC/ST	7761	26.40
OBC	9306	46.14
Others	7069	27.45
Religion		
Hindu	17,736	83.13
Muslim	2852	10.73
Others	3548	6.14
Place of residence		
Urban	8183	28.61
Rural	15,953	71.39
Region		
North	4496	12.72
Central	3369	21.75
East	4589	24.55
Northeast	2901	2.95
South	5758	22.14
West	3023	15.89
Total	24,136	100

*w col%* weighted column percentages to account for survey design and to provide national population estimates, *SD* standard deviation, *High IC* older adults with a score of greater than mean IC plus one SD (9 and above on a scale of 0–10), *ADL* activities of daily living, *IADL* instrumental activities of daily living, *MPCE* monthly per capita consumption expenditure.

**Figure 2:**
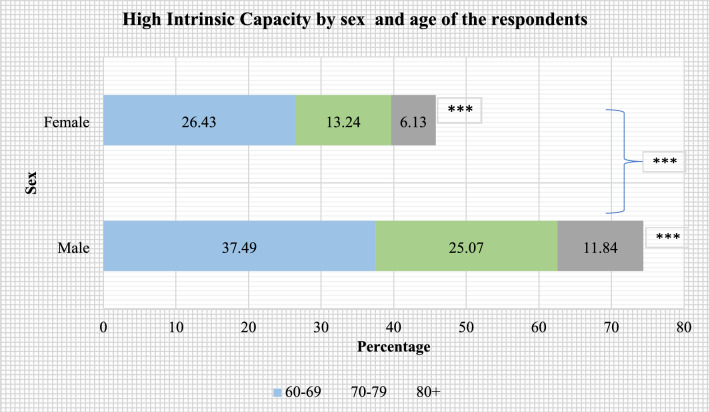
Percentage distribution of high intrinsic capacity among older adults, stratified by sex and age of the respondents (***: cross-tabulation showed statistically significant association at p-value < 0.001, based on Chi-square test).

**Figure 3:**
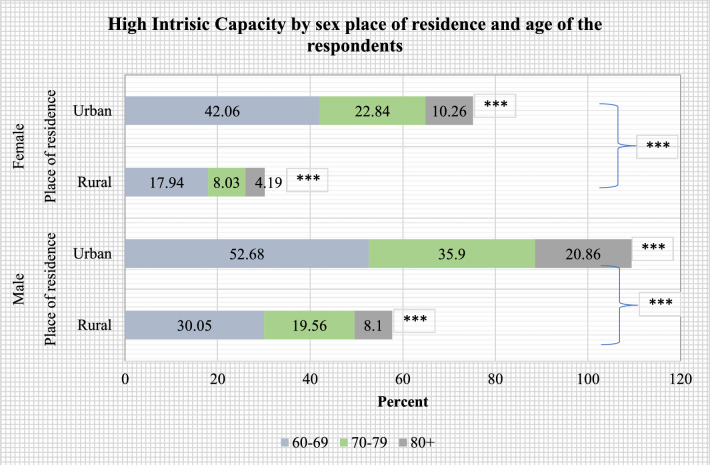
Percentage distribution of high intrinsic capacity among older adults, stratified by sex, place of residence and age of the respondents (***: cross-tabulation showed statistically significant association at p-value < 0.001, based on Chi-square test).

**Figure 4:**
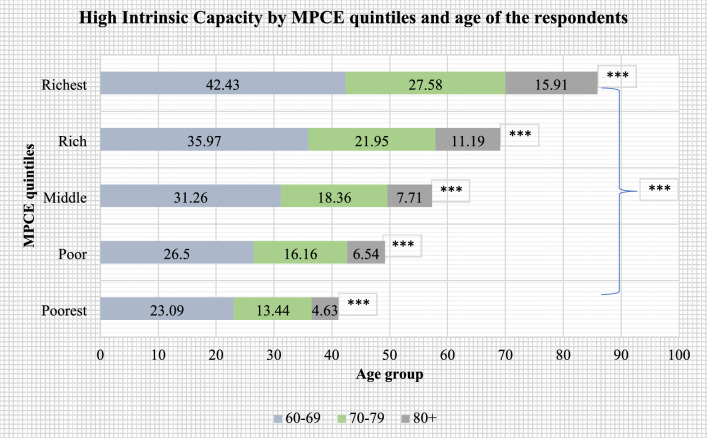
Percentage distribution of high intrinsic capacity among older adults, stratified by MPCE quintiles and age of the respondents (***: cross-tabulation showed statistically significant association at p-value < 0.001, based on Chi-square test).

**Figure 5:**
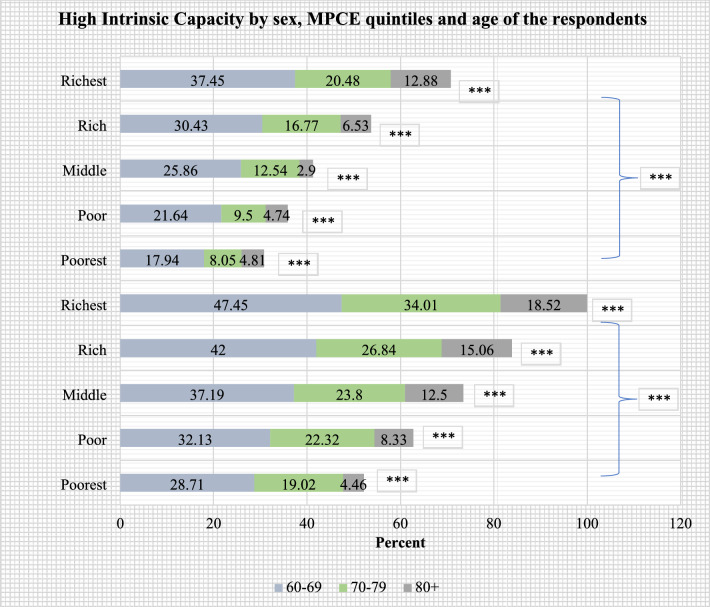
Percentage distribution of high intrinsic capacity among older adults, stratified by sex, MPCE quintiles and age of the respondents (***: cross-tabulation showed statistically significant association at p-value < 0.001, based on Chi-square test).

Figures 2, 3, 4, 5 present the stratified analysis of percentage distribution of high IC among older adults by sex, place of residence, MPCE quintiles and age of the respondents. It was found that older men (37.49%) and women (26.43%) who aged 60–69 years had a higher prevalence of high IC than their older counterparts. Male older adults aged 60–69 years who reside in urban areas had the highest prevalence of high IC (52.68%) in this study. A higher prevalence of high IC was also reported for older women aged 60–69 years who resided in urban areas (42.06%) than their rural-dwelling counterparts (17.94%). Older adults aged 80 + years who were from the richest wealth quintile (15.91%) had a higher prevalence of high IC than their poorest peers (4.63%). Also, older men aged 80 + years had a higher prevalence of high IC (18.52%) than their female peers from the richest quintile (12.88%).

Table 2 presents prevalence estimates of ADL difficulty, IADL difficulty, falls, multiple falls and fall-related injuries among older adults. The prevalence of ADL difficulty and IADL difficulty is estimated to be 19.89% and 45.00%, respectively. Prevalence of falls, multiple falls and fall-related injury is estimated to be 12.36%, 5.49% and 5.57%, respectively. Older adults who reported high IC had a lower prevalence of ADL difficulty (12.26% vs 22.38%) and IADL difficulty (31.13% vs 49.52%) than those who reported low IC. Similarly, a lower prevalence of falls (9.42% vs 13.34%), fall-related injury (4.10% vs 6.06%) and multiple falls (3.46% vs 6.16%) were reported among those who had high IC.

**Table 2:** Prevalence estimates of functional difficulty and falls among older adults.

Variables	ADL difficulty	IADL difficulty	Fall	Multiple falls	Fall-related injury
	w row % [95% CI]	w row % [95% CI]	w row % [95% CI]	w row % [95% CI]	w row % [95% CI]
High IC					
No	22.38 [21.36, 23.43]	49.52 [48.30, 50.74]	13.34 [12.55, 14.17]	6.16 [5.63, 6.74]	6.06 [5.51, 6.67]
Yes	12.26 [10.47, 14.30]	31.13 [26.68, 35.95]	9.42 [8.23, 10.76]	3.46 [2.89, 4.15]	4.10 [3.42, 4.91]
Age					
60–69	15.86 [14.91, 16.85]	39.07 [37.47, 40.69]	12.35 [11.49, 13.26]	5.3 [4.76, 5.90]	5.76 [5.18, 6.40]
70–79	23.7 [21.86, 25.65]	51.8 [49.17, 54.42]	11.84 [10.73, 13.05]	5.48 [4.73, 6.35]	4.93 [4.25, 5.72]
80 +	36.01 [31.40, 40.90]	64.68 [60.24, 68.89]	14.15 [11.52, 17.27]	6.87 [5.37, 8.74]	6.27 [4.37, 8.93]
Sex					
Male	17.73 [16.43, 19.12]	35.18 [33.67, 36.71]	11.27 [10.35, 12.27]	4.67 [4.10, 5.32]	5.04 [4.40, 5.76]
Female	21.93 [20.61, 23.31]	54.26 [52.29, 56.22]	13.43 [12.45, 14.47]	6.29 [5.67, 6.99]	6.1 [5.46, 6.81]
Marital status					
Currently in union	17.96 [16.85, 19.12]	38.64 [37.31, 39.99]	11.98 [11.14, 12.86]	5.15 [4.63, 5.72]	5.44 [4.87, 6.06]
Widowed	23.66 [21.88, 25.53]	56.71 [54.10, 59.28]	13.33 [12.13, 14.63]	6.3 [5.52, 7.19]	6.02 [5.22, 6.92]
Others	17.35 [13.04, 22.72]	47.6 [41.37, 53.90]	9.01 [6.18, 12.95]	3.23 [1.76, 5.86]	2.84 [1.49, 5.35]
Education					
No/primary	21.8 [20.74, 22.89]	50.91 [49.66, 52.15]	13.2 [12.40, 14.04]	6.08 [5.54, 6.66]	6.01 [5.44, 6.63]
Secondary	15.23 [13.43, 17.22]	32.76 [27.46, 38.53]	10.92 [9.39, 12.67]	4.62 [3.79, 5.61]	4.77 [3.89, 5.84]
Higher	13.94 [10.48, 18.30]	21.29 [17.57, 25.56]	8.4 [6.74, 10.43]	2.42 [1.60, 3.64]	3.65 [2.67, 4.97]
Work status					
Never worked	21.34 [19.37, 23.46]	51.19 [47.71, 54.66]	11.04 [9.79, 12.44]	5.22 [4.39, 6.19]	5.02 [4.25, 5.92]
Not working	25.06 [23.44, 26.76]	53.41 [51.59, 55.23]	13.05 [11.95, 14.24]	5.96 [5.25, 6.76]	5.6 [4.89, 6.40]
Working	13.15 [11.87, 14.54]	35.02 [33.28, 36.80]	13.47 [12.20, 14.84]	5.56 [4.80, 6.43]	6.44 [5.51, 7.52]
Retired	20.9 [17.05, 25.35]	29.05 [25.08, 33.37]	8.75 [6.99, 10.89]	3.94 [2.78, 5.55]	3.46 [2.56, 4.65]
Tobacco use					
No	19.59 [18.39, 20.86]	46.34 [44.45, 48.24]	11.89 [11.03, 12.81]	5.35 [4.81, 5.94]	5.71 [5.10, 6.39]
Yes	21.63 [19.88, 23.49]	45.01 [42.97, 47.06]	14.79 [13.32, 16.40]	6.49 [5.55, 7.58]	6.11 [5.22, 7.15]
Episodic alcohol					
No	20.17 [19.21, 21.16]	45.36 [43.97, 46.76]	12.36 [11.66, 13.10]	5.48 [5.03, 5.96]	5.59 [5.11, 6.10]
Yes	15.18 [11.85, 19.25]	38.85 [34.89, 42.96]	12.32 [10.06, 14.99]	5.67 [4.19, 7.64]	5.34 [3.93, 7.20]
Yoga					
No	20.23 [19.19, 21.31]	46.78 [45.29, 48.28]	11.93 [11.21, 12.70]	5.29 [4.83, 5.79]	5.41 [4.92, 5.96]
Yes	17.97 [16.24, 19.84]	34.86 [32.62, 37.16]	14.76 [13.00, 16.71]	6.64 [5.53, 7.95]	6.48 [5.37, 7.79]
SRH					
Very good	13.01 [8.25, 19.93]	23.9 [19.36, 29.11]	8.19 [5.86, 11.33]	2.05 [1.09, 3.81]	4.48 [2.88, 6.90]
Good	11.31 [9.81, 12.99]	33.28 [31.24, 35.39]	9.2 [8.15, 10.37]	3.05 [2.56, 3.63]	4.31 [3.62, 5.13]
Fair	19.44 [18.09, 20.86]	46.71 [44.40, 49.04]	12.62 [11.66, 13.65]	5.95 [5.30, 6.67]	5.65 [5.04, 6.33]
Poor	30.97 [28.83, 33.19]	59.16 [56.83, 61.46]	17.19 [15.29, 19.27]	8.47 [7.18, 9.96]	7.26 [5.90, 8.91]
Very poor	52.11 [45.60, 58.55]	69.33 [62.90, 75.09]	16.21 [11.83, 21.81]	9.9 [6.62, 14.54]	8.64 [5.46, 13.41]
Morbidity					
No	15.7 [14.51, 16.97]	40.05 [38.54, 41.59]	12.26 [11.26, 13.33]	5.31 [4.69, 6.02]	5.36 [4.69, 6.12]
Single	20.3 [18.69, 22.01]	46.06 [44.01, 48.12]	12.25 [11.12, 13.47]	5.07 [4.38, 5.85]	5.49 [4.72, 6.37]
Two	26.64 [24.16, 29.28]	49.27 [46.35, 52.20]	12.09 [10.59, 13.78]	5.69 [4.69, 6.88]	5.63 [4.65, 6.80]
Three plus	30.98 [24.80, 37.92]	62.97 [55.18, 70.13]	14.11 [11.04, 17.87]	8.06 [5.97, 10.78]	7.29 [5.29, 9.95]
Wealth quintile					
Poorest	20.99 [19.17, 22.95]	46.85 [44.56, 49.16]	10.93 [9.73, 12.27]	5.1 [4.28, 6.07]	4.5 [3.75, 5.40]
Poorer	19.98 [18.10, 21.99]	45.71 [43.47, 47.96]	13.3 [11.78, 14.98]	5.88 [4.88, 7.09]	5.45 [4.48, 6.62]
Middle	20.65 [18.44, 23.04]	43.38 [40.77, 46.04]	11.48 [10.05, 13.09]	5.02 [4.23, 5.95]	5.84 [4.74, 7.17]
Richer	18.45 [16.56, 20.50]	45.19 [41.52, 48.92]	12.94 [11.31, 14.78]	5.93 [4.92, 7.14]	5.37 [4.43, 6.49]
Richest	19.11 [16.86, 21.59]	43.5 [39.28, 47.81]	13.44 [11.91, 15.13]	5.53 [4.59, 6.65]	7.09 [5.97, 8.41]
Religion					
Hindu	19.79 [18.74, 20.88]	44.99 [43.46, 46.54]	12.4 [11.65, 13.20]	5.5 [5.02, 6.03]	5.62 [5.10, 6.18]
Muslim	21.65 [19.33, 24.16]	45.42 [42.41, 48.46]	12.42 [10.45, 14.71]	5.31 [4.19, 6.71]	5.66 [4.44, 7.18]
Others	18.28 [15.53, 21.39]	44.38 [41.07, 47.75]	11.61 [9.77, 13.73]	5.56 [4.38, 7.02]	4.79 [3.61, 6.33]
Caste					
SC/ST	20.46 [18.82, 22.19]	45.31 [43.33, 47.30]	12.5 [11.28, 13.82]	4.95 [4.23, 5.79]	6.13 [5.25, 7.14]
OBC	19.18 [17.66, 20.81]	47.42 [45.04, 49.81]	11.66 [10.65, 12.74]	5.6 [4.96, 6.32]	4.91 [4.25, 5.68]
Others	20.55 [19.15, 22.02]	40.65 [38.89, 42.43]	13.38 [12.12, 14.76]	5.8 [4.98, 6.75]	6.13 [5.31, 7.05]
Place of residence					
Urban	17.29 [15.52, 19.23]	37.05 [33.27, 40.99]	9.67 [8.61, 10.85]	4.35 [3.68, 5.13]	4.58 [3.84, 5.45]
Rural	20.94 [19.89, 22.02]	48.19 [46.98, 49.40]	13.44 [12.62, 14.31]	5.95 [5.42, 6.52]	5.97 [5.41, 6.58]
Region					
North	11.09 [9.96, 12.34]	38.41 [36.46, 40.40]	10.2 [9.01, 11.53]	4.71 [3.85, 5.75]	4.7 [3.84, 5.73]
Central	17.27 [15.31, 19.42]	40.04 [37.67, 42.45]	13.81 [12.19, 15.60]	7.28 [6.09, 8.68]	5.79 [4.75, 7.04]
East	25.21 [23.31, 27.21]	47.95 [45.84, 50.07]	14.64 [13.19, 16.21]	6.03 [5.23, 6.94]	7.05 [6.02, 8.23]
Northeast	14.36 [12.39, 16.59]	38.45 [35.67, 41.30]	9.71 [8.02, 11.72]	4.28 [3.19, 5.73]	4.52 [3.37, 6.04]
West	16.41 [14.30, 18.76]	53.35 [49.27, 57.38]	8.82 [7.67, 10.12]	4.38 [3.63, 5.27]	2.94 [2.36, 3.66]
South	28.21 [25.86, 30.69]	42.08 [39.38, 44.83]	14.15 [12.35, 16.15]	4.64 [3.73, 5.76]	7.62 [6.28, 9.21]
Total	19.89 [18.97, 20.85]	45.00 [43.67, 46.34]	12.36 [11.68, 13.06]	5.49 [5.06, 5.95]	5.57 [5.11, 6.07]

*w row %* weighted row percentages by using svyset command to account for complex survey design and provide population estimates, *CI* confidence interval, *ADL* activities of daily living, *IADL* instrumental activities of daily living, *SRH* self-rated health, *MPCE* monthly per capita consumption expenditure.

**Figure 6:**
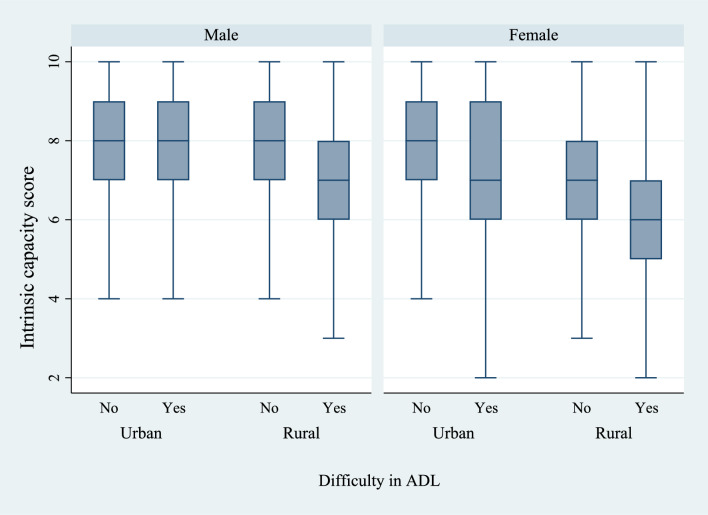
Box plot of high intrinsic capacity by difficulty in ADL among older adults, stratified by sex and place of residence.

**Figure 7:**
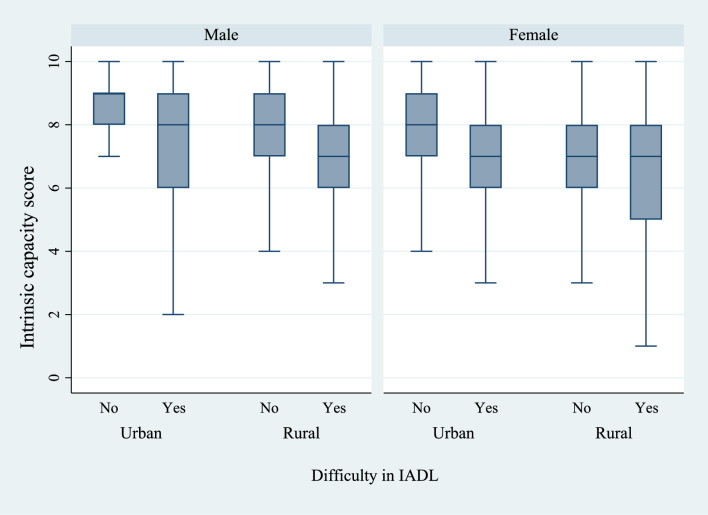
Box plot of high intrinsic capacity by difficulty in IADL among older adults, stratified by sex and place of residence.

**Figure 8:**
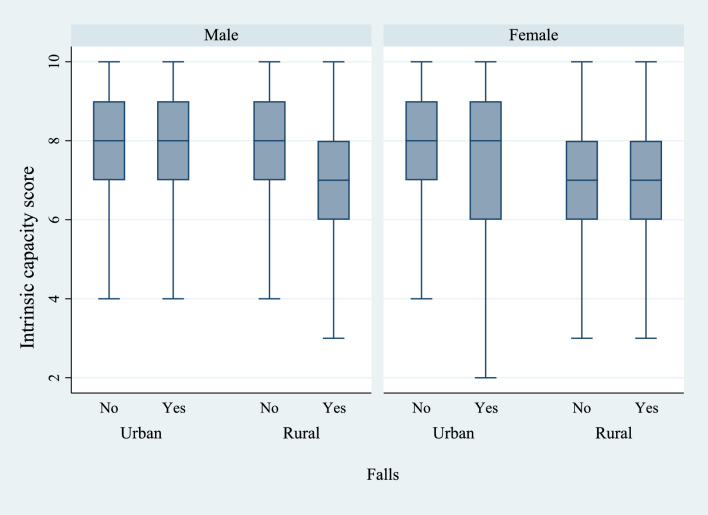
Box plot of high intrinsic capacity by falls among older adults, stratified by sex and place of residence.

**Figure 9:**
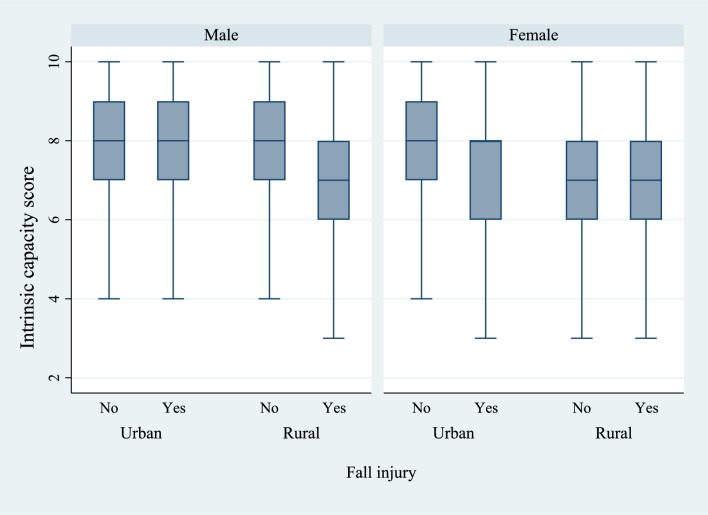
Box plot of high intrinsic capacity by fall injury among older adults, stratified by sex and place of residence.

**Figure 10:**
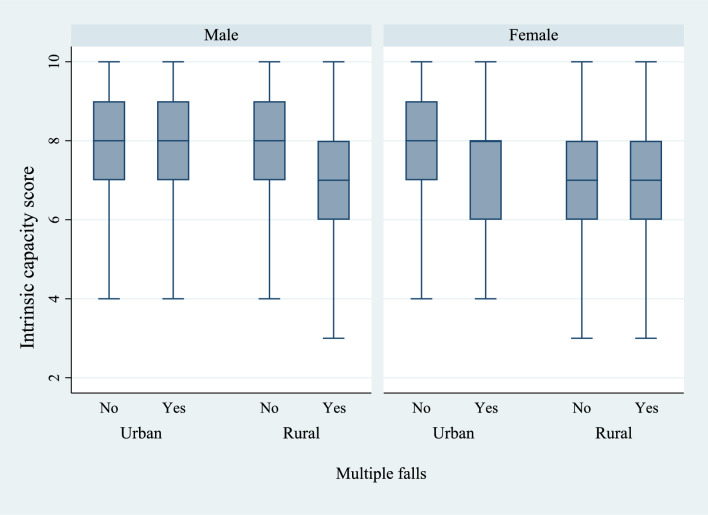
Box plot of high intrinsic capacity by multiple falls among older adults, stratified by sex and place of residence.

Figures 6 and 7 present the box plots of intrinsic capacity score by the prevalence of difficulties in ADL and IADL, stratified by sex and place of residence. There was a significantly higher prevalence of functional difficulties among older women who lived in rural regions of the country and who had a lower IC score. Figures 8, 9, and 10 present the box plots of intrinsic capacity score by the prevalence of fall outcomes stratified by sex and place of residence. Older women living in rural India with a lower IC score reported higher rates of fall, multiple falls, and fall injuries in this study.

Table 3 presents adjusted odds ratios from logistic regression of functional difficulty and falls by high IC and other socioeconomic and lifestyle factors among older adults. Older adults with high IC had significantly lower odds of ADL difficulty [aOR: 0.63, CI: 0.52–0.76], IADL difficulty [aOR: 0.71, CI: 0.60–0.83], falls [aOR: 0.80, CI: 0.67–0.96], multiple falls [aOR: 0.73, CI: 0.58–0.96] and fall-related injury [aOR: 0.78, CI: 0.61–0.99]. Older adults with multimorbidity had significantly higher odds of ADL difficulty [aOR: 2.12, CI: 1.66–2.71], IADL difficulty [aOR: 2.69, CI: 1.85–3.9] and fall-related injury [aOR: 1.49, CI: 1.03–2.15]. Similarly, females had significantly higher odds of functional difficulty and falls. Further, significantly lower odds of functional difficulty and falls were reported for participants with higher education and who were working.

**Table 3:** Adjusted odds ratios from logistic regression of disability and falls by high IC and other socioeconomic and lifestyle factors among older adults.

Variables	ADL difficulty	IADL difficulty	Falls	Multiple falls	Fall-related injury
High IC					
No	Ref.	Ref.	Ref.	Ref.	Ref.
Yes	0.63*** (0.52–0.76)	0.71*** (0.60–0.83)	0.80* (0.67–0.96)	0.73** (0.58–0.92)	0.78* (0.61–0.99)
Age (in years)					
60–69	Ref.	Ref.	Ref.	Ref.	Ref.
70–79	1.42*** (1.23–1.64)	1.45*** (1.25–1.67)	0.93 (0.80–1.09)	0.94 (0.75–1.17)	0.81 (0.66–1.01)
80 +	2.38*** (1.75–3.23)	2.20*** (1.62–2.99)	1.14 (0.87–1.51)	1.27 (0.93–1.75)	1.09 (0.70–1.71)
Sex					
Male	Ref.	Ref.	Ref.	Ref.	Ref.
Female	1.18* (1.00–1.39)	1.76*** (1.54–2.02)	1.37** (1.15–1.64)	1.59*** (1.22–2.05)	1.14 (0.88–1.48)
Marital status					
Currently in union	Ref.	Ref.	Ref.	Ref.	Ref.
Widowed	1.02 (0.87–1.18)	1.26** (1.10–1.45)	1.08 (0.93–1.25)	1.08 (0.88–1.32)	1.06 (0.85–1.31)
Others	0.82 (0.54–1.24)	1.16 (0.84–1.59)	0.82 (0.51–1.31)	0.67 (0.34–1.32)	0.43* (0.19–0.99)
Education					
No/primary	Ref.	Ref.	Ref.	Ref.	Ref.
Secondary	0.73*** (0.60–0.89)	0.62*** (0.51–0.75)	0.94 (0.76–1.16)	0.88 (0.67–1.15)	0.85 (0.64–1.12)
Higher	0.65* (0.45–0.93)	0.50*** (0.38–0.65)	0.71* (0.52–0.98)	0.52*** (0.33–0.82)	0.53*** (0.35–0.81)
Work status					
Never worked	Ref.	Ref.	Ref.	Ref.	Ref.
Not working	1.10 (0.94–1.29)	1.22* (1.05–1.43)	1.38*** (1.14–1.66)	1.40** (1.08–1.81)	1.23 (0.95–1.59)
Working	0.69*** (0.57–0.84)	0.84* (0.72–0.99)	1.75*** (1.44–2.14)	1.68*** (1.27–2.23)	1.74*** (1.32–2.29)
Retired	1.64** (1.18–2.27)	1.04 (0.77–1.39)	1.19 (0.87–1.65)	1.39 (0.88–2.18)	1.09 (0.72–1.65)
Tobacco use					
No	Ref.	Ref.	Ref.	Ref.	Ref.
Yes	1.05 (0.91–1.21)	1.10 (0.98–1.24)	1.12 (0.94–1.32)	1.16 (0.92–1.47)	0.85 (0.66–1.09)
Episodic alcohol drinking					
No	Ref.	Ref.	Ref.	Ref.	Ref.
Yes	0.95 (0.62–1.46)	1.04 (0.81–1.32)	1.21 (0.88–1.66)	1.50* (1.00–2.25)	1.12 (0.71–1.75)
Yoga					
No	Ref.	Ref.	Ref.	Ref.	Ref.
Yes	0.98 (0.82–1.17)	0.88 (0.77–1.02)	1.45*** (1.20–1.74)	1.45** (1.15–1.83)	1.16 (0.92–1.48)
SRH					
Very good	Ref.	Ref.	Ref.	Ref.	Ref.
Good	0.77 (0.43–1.36)	1.31 (0.94–1.83)	1.10 (0.72–1.68)	1.36 (0.67–2.74)	0.91 (0.54–1.55)
Fair	1.28 (0.74–2.21)	1.99*** (1.43–2.77)	1.45* (0.96–2.19)	2.49** (1.24–4.99)	1.15 (0.69–1.92)
Poor	2.15*** (1.22–3.77)	2.57*** (1.82–3.61)	2.06*** (1.33–3.20)	3.19*** (1.54–6.58)	1.55 (0.87–2.77)
Very poor	4.45*** (2.34–8.46)	3.47*** (2.16–5.57)	1.97* (1.08–3.59)	3.81*** (1.62–8.99)	2.14 (0.98–4.66)
Morbidity					
No	Ref.	Ref.	Ref.	Ref.	Ref.
Single	1.21* (1.04–1.42)	1.20** (1.06–1.36)	1.03 (0.87–1.21)	0.93 (0.74–1.16)	1.14 (0.90–1.44)
Two	1.64*** (1.38–1.96)	1.34*** (1.15–1.55)	1.03 (0.84–1.26)	1.11 (0.85–1.45)	1.17 (0.88–1.56)
Three plus	2.12*** (1.66–2.71)	2.69*** (1.85–3.92)	1.15 (0.87–1.52)	1.36 (0.97–1.93)	1.49* (1.03–2.15)
MPCE quintile					
Poorest	Ref.	Ref.	Ref.	Ref.	Ref.
Poorer	1.00 (0.83–1.20)	1.07 (0.92–1.24)	1.25* (1.01–1.55)	1.20 (0.89–1.63)	1.30 (0.95–1.77)
Middle	1.18 (0.96–1.44)	1.01 (0.86–1.20)	1.17 (0.94–1.46)	1.12 (0.84–1.51)	1.53* (1.11–2.11)
Richer	1.01 (0.83–1.23)	1.05 (0.87–1.28)	1.28* (1.02–1.59)	1.23 (0.91–1.66)	1.31 (0.96–1.79)
Richest	1.10 (0.87–1.39)	1.04 (0.84–1.28)	1.51*** (1.21–1.89)	1.27 (0.93–1.74)	2.16*** (1.59–2.95)
Religion					
Hindu	Ref.	Ref.	Ref.	Ref.	Ref.
Muslim	0.99 (0.82–1.18)	0.96 (0.82–1.13)	0.93 (0.74–1.18)	0.87 (0.64–1.20)	0.88 (0.64–1.21)
Others	1.01 (0.80–1.29)	1.01 (0.84–1.22)	1.03 (0.81–1.31)	1.25 (0.92–1.70)	0.79 (0.55–1.14)
Caste					
SC/ST	Ref.	Ref.	Ref.	Ref.	Ref.
OBC	0.92 (0.78–1.08)	1.15* (1.01–1.31)	1.07 (0.89–1.29)	1.40** (1.09–1.80)	0.89 (0.69–1.16)
Others	1.00 (0.84–1.18)	1.06 (0.91–1.23)	1.29* (1.06–1.58)	1.64*** (1.23–2.18)	1.05 (0.80–1.38)
Place of residence					
Urban	Ref.	Ref.	Ref.	Ref.	Ref.
Rural	1.28** (1.10–1.48)	1.63*** (1.43–1.87)	1.22* (1.03–1.44)	1.17 (0.94–1.46)	1.09 (0.86–1.39)
Region					
North	Ref.	Ref.	Ref.	Ref.	Ref.
Central	1.88*** (1.49–2.36)	1.10 (0.93–1.29)	1.48** (1.18–1.87)	1.80*** (1.32–2.45)	1.35 (0.98–1.86)
East	2.75*** (2.24–3.37)	1.38*** (1.18–1.60)	1.61*** (1.31–1.98)	1.45** (1.10–1.91)	1.68*** (1.26–2.25)
Northeast	1.66*** (1.28–2.15)	1.06 (0.88–1.27)	0.98 (0.73–1.31)	1.02 (0.68–1.53)	1.14 (0.76–1.71)
West	1.54*** (1.22–1.95)	1.72*** (1.43–2.07)	0.87 (0.68–1.11)	0.95 (0.68–1.33)	0.58*** (0.41–0.82)
South	4.20*** (3.37–5.24)	1.44*** (1.21–1.70)	1.56*** (1.22–2.00)	1.07 (0.76–1.51)	1.87*** (1.36–2.58)
Constant	0.05*** (0.03–0.10)	0.12*** (0.08–0.18)	0.03*** (0.02–0.05)	0.01*** (0.00–0.02)	0.02*** (0.01–0.05)

* if p-value < 0.05, ** if p-value < 0.005, and *** if p-value < 0.001; *aOR* OR adjusted for all the covariates, *ADL* activities of daily living, *IADL* instrumental activities of daily living, *MPCE* monthly per capita consumption expenditure.

## Discussion

Research on the health relevance of IC for older adults is at the nascent stage in India, although IC has been widely studied in several other countries across the globe. As such, using a large nationally representative survey data, the present study examined the prevalence and correlates of high IC and its association with five adverse health outcomes, including ADL difficulty, IADL difficulty, fall, multiple falls, and fall-related injury among older adults in India. Given that the IC model is proven to be highly promising for improving the future medical approach, our contributions carry important practical implications for geriatric health care and healthy aging policy formulation. Our study demonstrated that after adjusting for socio-demographic, behavioral characteristics and morbidity, high IC was independently associated with a lower risk of functional difficulty and fall outcomes in later life. Taken together our findings imply that because regular IC monitoring can predict poor health outcomes in older adults, improvement in IC should be prioritized while formulating disability and fall prevention strategies.

A considerably higher proportion of older adults reported ADL difficulty (19.89%) and IADL difficulty (45%) in line with previous studies conducted in India^31,60^. The higher prevalence of functional disability corroborates the argument of WHO that the prevalence of disability among older adults is increasing drastically due to the combined effect of demographic and epidemiological transitions^61^. Further, in the past 2 years, a significant percentage of older adults reported falling (12.36%), which is lesser than the estimates projected by research conducted in other nations^49^. The pooled prevalence of falls was found to be 31% in a systematic review and meta-analysis of the burden of falls among older Indians^27^. This disparity in the prevalence of falls could be attributed to the fact that the majority of studies have relied on data from self-reported falls. Consequently, there remains the possibility of underestimation or overestimation of the burden of falls due to recall bias^27^. Nevertheless, the prevalence of falls, multiple falls, and fall-related injuries in the present study were consistent with prior evidence from India^49,62^.

Consistent with previous studies conducted in developing and developed countries, our study revealed that individuals enjoying high IC report lower odds of functional difficulty measured in ADLs and IADLs^11,12,14^. The predictive validity of composite IC scores has been confirmed in both cross-sectional and longitudinal investigations^11,23^. Evidence from the literature shows that biomarkers such as phase angle, grip strength (vitality), and gait speed (locomotion) as IC indicators significantly predict IADL difficulty, but not other evaluated outcomes, namely ADL difficulty and frailty^23^. Also, a decline in functional performance is linked with lower nutritional status^63^. Similarly, the intensity of depressive symptoms, which is an indicator of psychological health^64^ and lower cognitive performance evaluated by MMSE^14^ are linked to eventual physical decline among older adults. Physical decline, which is defined as a decrease in muscle strength and reduced mobility, has frequently been recognized as an "additional vital sign" for older adults and a crucial element of the geriatric assessment^65,66^. Thus, monitoring IC trajectories would effectively prevent physical decline and capture the onset of ADL and IADL difficulties. Additionally, multi-component interventions such as nutrition supplements, physical activity promotion and depression management may also improve IC and, thereby, functional performance^67^.

The current study also observed that a high IC score is significantly associated with lower odds of falls and multiple falls after adjusting for the impact of potential confounders such as age, sex, health-related attributes and lifestyle behaviors. The study evaluating the predictive value of the domains of IC on the adverse health events among nursing home residents suggested that lower scores in subdomains of IC, namely, balance, nutrition, gait speed, chair stand performance and handgrip strength, increased the risk of falls and multiple falls during 3 years follow up period^15^. A recent Chinese study also emphasised that IC decline was independently associated with higher risks of frailty, disability, falls, fractures and immobility^9^. Our finding is consistent with the evidence from these cross-sectional^9^ and longitudinal studies^15^. As evidence suggests, interventions focused on improving IC scores must target reducing the risk of falls and multiple falls among community-dwelling older adults.

We also discover that fall-related injury in old age can be associated with IC score. Older adults with higher IC had a lower incidence of fall-related injury in the late-life, which is consistent with the finding that IC decline is significantly associated with increased odds of falls and fractures among community-dwelling older adults^9^. Falls, likely, result in severe injuries such as fractures, cerebral haemorrhage and death. Fall-related injuries, in turn, result in longer hospital stays and higher medical costs^68^. The connection between distinct body functions, which comprise the IC domains, and fall-related injury is established in earlier studies. For instance, research demonstrates that issues with movement, balance, and muscular wasting increase the risk of falling^69^. Multiple studies have also proven that loss of balance and drowsiness elevate the risk of falling in older adults^70,71^. Similarly, self-reported walking difficulty, complete or partial vision impairment and cognitive impairment have emerged as separate risk factors for fall-related injuries in older Indians^56^.

In contrast with earlier studies, the present study shows that there is no statistically significant difference in the prevalence of fall-related injuries among older men and women^55,56^. Though, consistent with the literature, education is protective against fall-related injury. Individuals with higher educational levels are less likely to fall and experience injuries that result in death. This could be partially explained by the lack of resources for understanding injury prevention^72^. In this regard, intervention through health education can be worthwhile for mitigating fall-related outcomes. The findings also indicated that fall-related injury was significantly associated with those who reported poor self-rated health and multimorbidity in support of a Malaysian epidemiological study that investigated the risk factors of falls^73^. Mirroring the findings in studies in India, the present study revealed that higher socioeconomic status is protective against falls and fall-related injury^56,74^. Further, the southern states were found to have a higher chance of fall-related injury, suggesting that fall prevention strategies must be designed after identifying the various fall risks that older populations in various states face.

Moreover, recent Indian studies have explored sex differences in functional health outcomes^55,56,75,76^. The present study's findings also corroborate the female disadvantage in functional difficulty and fall outcomes observed in previous studies. Women's weaker quadriceps muscles and declining bone density compared to males, especially after menopause, may contribute to the sex differences^77^. Also, the fact that women live longer than men and consequently are more likely to experience adverse health events as they age can explain these sex differences^78^. The finding of our study also shows a significant rural–urban disparity following prior evidence^75^. Specifically, older adults dwelling in rural India reported higher rates of ADL and IADL difficulties, falls, multiple falls and fall injuries. The poor functional status of rural residents may reflect inadequate health and health care infrastructure^79^. Our findings, thus, suggest that interventions to improve functional health should focus on diverse groups of older adults, namely older women, rural older adults, and those with fewer socioeconomic resources for self-care.

The findings of our study must be interpretated within the context of certain limitations. *First*, the cross-sectional nature of these data precludes us from staking any predictive claims. Future work using forthcoming waves of LASI may render a more conclusive statement on the associations between IC and functional vitality among older Indians. Because the concept of IC engages a comprehensive approach to one’s functional status across the life course, a longitudinal analysis of an individual’s IC trajectory may offer greater opportunities for early intervention to maintain functional potency in later ages^6^. *Second*, given the lack of agreement on how to measure IC, either in terms of the indicator selection, or how it is calculated, weighted, or validated, inferences based on our findings remain limited. Similarly, the lack of information on several measures of specific indicators, such as vestibular and somatosensory system in relation to sensory capacity, may limit the interpretation of our findings. *Third*, the components of sensory and psychological domains of IC, in the current study were based on self-report, which may have been influenced by reporting and recall biases. Future studies may want to consider using objective tests of functioning, especially when it comes to certain sensory domains, namely vision and hearing related impairments.

*Fourth*, older people with a higher BMI were classified as having greater vitality score. This was based on the rationale that older individuals who are overweight/obese may be a select segment of the population who had averted the otherwise anticipated negative consequences of excess weight on health. Moreover, gradual age-related loss in body height may have resulted in overestimation of BMI and a higher BMI among older adults is not always associated with adverse health outcomes^80–82^. In the current analysis, we also controlled for the effects of chronic diseases and lifestyle behaviors which could adjust the potential negative influence of comorbidities related to obesity in the association between IC and health outcomes. Future studies using upcoming waves of the LASI data may permit the assessment of the impact of changes in BMI on changes in vitality among older Indians.

*Fifth,* instead of a weighted score, we used a composite IC score. Future studies may want to consider alternative statistical approaches to calculate IC scores. The present study was not able to analyze crucial behavioral factors that contribute to the risk for falling, such as dietary patterns, and genetic factors that may influence the IC of older adults, which need to be considered in future research. *Finally*, there still is no universally accepted definition or consensus on what constitutes a fall. Studies vary widely in how a fall is defined, and there also remain differences in the timeframe used to gauge the incidence of falls (e.g., within the past year or less versus 2 years). These discrepancies, in consequence, preclude any direct comparison of findings across existing studies^83^.

Notwithstanding these limitations, our study has several strengths. This study is only the second of its kind to examine IC and its association with selected adverse health outcomes in India's aging population using a large, nationally representative sample. Relatedly, the data we used allowed us to assess the relevance of several conceptually relevant social, demographic, and lifestyle factors. Doing so in a resource restrained LMIC like India is worth noting. Further, most IC components in our study are assessed based on unified performance tests or anthropometric measures, which prevent response or interviewer bias.

## Conclusion

The present study found that after adjusting for potential confounders such as age, sex, health-related attributes and lifestyle behaviors, a high IC is independently associated with a lower risk of functional difficulty and fall outcomes among older Indians. Our findings support the strategy of optimizing IC in pursuit of healthy aging and underscore the need for creating an IC care cascade, especially for older adults who are socially and economically vulnerable. Such an intervention, we believe, may prove consequential for families as well as they prepare to care for their older kin. To prevent or impede IC deficits and to mitigate its association with functional deficits, including falls and falls related injuries, it is crucial to assess IC holistically^38^ and the first important step in this process is to train health professionals in administering IC screening to older adults. This step is particularly critical in India where socioeconomic mobility is low, affordable health care is out of reach for many, and systems of formal long-term care are feeble at best^84–86^. Formal IC screening in such a context would both, help identify those most susceptible to functional decline and stimulate more formal and informal systems of support to prevent and prolong such functional decline.

## Data Availability

The datasets used and/or analysed during the current study are available in the repository of the Gateway to Global Aging Data (https://g2aging.org/).
